# Insights in the treatment of congenital nasolacrimal duct obstruction

**DOI:** 10.22336/rjo.2017.19

**Published:** 2017

**Authors:** Elena Avram

**Affiliations:** * Ophthalmology Department, Medlife Băneasa Hyperclinic, Bucharest, Romania

**Keywords:** congenital nasolacrimal duct obstruction, probing, dacryocystorhinostomy

## Abstract

**Introduction:** Congenital nasolacrimal duct obstruction is one of the most common causes of epiphora in newborns and the main cause of this condition is the persistence of Hasner membrane. Several treatment options are available, like conservative treatment, probing, irrigation, or more complex techniques.

**Objective:** The objective of this paper is to discuss the efficiency of different treatment options addressing congenital nasolacrimal duct obstruction based on trials reported in literature.

**Methods:** Clinical trials were identified on PubMed. The results were discussed regarding patient age, type of treatment and efficiency of the treatment.

**Results:** 41 trials were reviewed. The rate of resolution according to different treatment options was the following: conservative treatment 14.2-96%, probing 78-100%, irrigation 33-100%, silicon tube intubation 62-100%, inferior turbinate fracture 54.7-97%, balloon dacryocystoplasty 77%, endoscopic intranasal surgery 92.72%, and dacryocystorhinostomy 88.2-93.33%.

**Conclusions:** The first choice in uncomplicated cases should be a conservative treatment, which can be followed until the age of 1 year, while in complicated cases other solutions should be considered.

**Abbreviations:** CNDO = Congenital nasolacrimal duct obstruction, DCR = Dacryocystorhinostomy, MCI = Monocanalicular intubation, BCI = Bicanalicular intubation

## Introduction

In the management of congenital nasolacrimal duct obstruction (CNDO), very complex and noninvasive treatment options can be used: conservative treatment, probing, irrigation, silicon tube intubation, inferior turbinate fracture, balloon dacryocystoplasty, endoscopic intranasal surgery, or dacryocystorhinostomy. It is important that the technique we choose to be in correlation with the etiology of the disease and the complexity of the case. 

## Objective

The objective of this paper was to analyze the efficiency of different treatment options addressing CNDO, based on trials reported in literature.

## Methods

Clinical trials were identified on PubMed. The results were discussed regarding patient age, type of treatment and efficiency of treatment. 

## Results 

**• Conservative treatment**

At present, the current trend is to indicate a conservative treatment and expect spontaneous remission of CNDO. Conservative treatment entails: lacrimal sac compression and massage, rigorous hygiene of the eyelids and, if there is any purulent discharge, antibiotic eye drops (netilmicin). It is generally recommended up to the age of 12 months and then, depending on the severity of the symptoms, other therapeutic options can be discussed. The success rate is between 14.2% and 96% depending on the patients’ age (**Table 1**). It seems that compliance to treatment is a key factor. Karti compared two groups, one in which parents regularly applied lacrimal sac massage with a remission of 92.2% and another group with parents who did not regularly apply lacrimal sac massage, with a success rate of 77.7% of the cases [**3**].

**Table 1:** Success rate of conservative treatment during the first 12 months of life [**1**,**2**]

Patient age	Kakizaki H (2008)	PEDIG (2012)
1 month	82.9%	-
2 months	82.4%	-
3 months	80%	-
4 months	79.3%	-
5 months	76%	-
6 months	68.4%	-
7 months	66.7%	69%
8 months	64.7%	68%
9 months	57.1%	55%
10 months	33.3%	67%
11 months	14.2%	-

** • Probing**

This method has long been considered the first choice treatment in CNDO and it can be performed under local anesthesia before the age of 4-6 months or under general anesthesia [**4**,**5**].

The right moment of probing remains controversial, the main problem being the possibility of spontaneous resolution during the first 12 months of life. However, it is advisable to be performed before the age of 12 months if complications appear.

Some physicians prefer to approach the nasolacrimal duct from the upper punctum while others from the lower punctum or from both sides. The punctum is dilated. The probe is introduced vertically. The lid is pulled laterally and the probe is advanced horizontally until it reaches the nasal wall of the lacrimal sac. The lateral traction is released and the probe is turned 90 degrees and directed downward, posteriorly and laterally. 

There are several probing techniques:

– Probing guided with soft cannula which implies that a plastic intravenous catheter sheath is supported intraluminal with a guiding metal probe and has a success rate of 89.8% [**6**]; 

– Probing with manually bent Bowman probes that mimic the natural curve of the nasolacrimal duct have a success rate of 91.4%, while straight Bowman probes have a success rate of 76.2% [**7**];

– Endoscopic assisted probing allows a direct visualization of the nasolacrimal duct and avoids the formation of false routes, its efficiency varying between 92.3% and 100% [**8**-**10**].

Success rate of probing varies between 78 and 100% (**Table 2**) and decreases with the age of the patient. Takahashi showed that the success rate in the second probing is lower than in the first [**14**]. 

Main side effects of this therapeutic approach are creating false routes and epithelium damage due to scarring strictures tear.

**Table 2:** Success rate after probing [**4**,**5**,**11**-**13**]

	Patients’ age	Success rate	Type of anesthesia
Abrishami M (2009)	15 – 24 months	76%	general
	25 – 36 months	67.7%	
	37 – 48 months	90%	
	49 – 60 months	60%	
Arora S (2012)	> 60 months	75%	general
	< 36 months	78%	
	> 36 months	50%	
Rajabi MT (2014)	24 – 36 months	85%	general
	37 – 48 months	63%	
	49 – 60 months	50%	
	< 6months	90.1%	
Hung CH (2015)	6 – 11 months	79.6%	topical
	12 – 17 months	76.8%	
	18 – 23 months	73.5%	
	24 – 35 months	75%	
	36 – 60 months	33%	
Le Garrec J (2016)	< 12 months	76.7%	topical

**• Irrigation**

This technique, with an efficiency of 33 - 100% according to various authors, involves injecting saline solution with or without antibiotic in the lacrimal pathways and is considered less invasive than probing. It can affect the tear ducts epithelium and can lead to the pulmonary aspiration of the fluids used [**15**,**16**].

**• Silicon tube intubation**

Lacrimal pathways prosthesis with silicone tubes is indicated in ineffective conservative treatment, failed probing, or presence of strictures.

There are two main types of silicone tubes used: monocanalicular (Mono Crawford, Monoka, Masterka) and bicanalicular (Crawford, Bika, Infant - Bika, Goldberg, Ritleng) and it is recommended that this invasive maneuver is performed under endoscopic control.

According to various authors, the success rate is between 62% and 100% [**17**-**26**]. Also Kassif showed that the rate of spontaneous resolution after unsuccessful intubation with silicone tube is 80% [**26**]. 

Several studies in which monocanalicular intubation (MCI) with bicanalicular intubation (BCI) results were compared were published. Some scientists like Rajabi showed that BCI is more effective while others, like Lee or Komínek, did not find a significant difference between the two groups [**19**,**23**,**24**]. Also regarding MCI, Andalib and Rajabi reported better results with Monoka stent than with Masterka stent [**24**,**25**].

Complications encountered are: symptoms relapse due to premature removal, atony of lacrimal punctum, corneal erosions, displacement, injury to the nasal mucosa and lower cone during the recovery procedure of the dislodged silicone tube [**17**-**26**].

**• Inferior turbinate fracture**

The use of inferior turbinate fracture usually associated with probing is recommended when there is a narrow space around the nasolacrimal duct ostium. 

This technique has a controversial efficiency. Ab. Attarzadeh and Katowitz did not found a high rate of success while Havins reported no failure when applying inferior nasal conchae fracture [**27**-**29**].

**• Balloon dacryocystoplasty**

Dilatation with balloon catheter or balloon dacryocystoplasty is performed by inserting a guide wire with a deflated balloon attached through the punctum in the nasolacrimal duct. The balloon is gently inflated with liquid and the pressure created opens up and expands the blocked duct. The balloon is deflated and removed. The success rate is 77% [**30**]. 

Hu compared balloon catheter dilatation with silicon intubation after a failed probing and obtained a 64.7% remission rate in the first group and an 86.1% remission rate in the second group [**31**]. This method is beginning to be proposed as an alternative to silicone tube intubation, having a lower rate of complications like epistaxis and lacrimal duct laceration [**32**].

**• Endoscopic intranasal surgery**

Proposed by Korkmaz, this new technique consists in an endoscope-guided inspection of the Hasner valve area, irrigation, incision of the imperforate Hasner membrane valve and again irrigation. The success rate is 92.72% [**33**].

**• Dacryocystorhinostomy**

Dacryocystorhinostomy (DCR) involves creating an anastomosis between the lacrimal sac and the nasal mucosa by means of a localized bone resection of the nose wing. It represents the last resort when other therapeutic methods have failed.

There are two types of DCR, external and internal (endonasal/ endoscopic). The success rate of the two surgical approaches in children is relatively the same (**Table 3**). Choung indicated that combining DCR with silicone tube intubation prevents failure, while Pakdel showed that this technique is not superior [**38**,**39**].

Kamal published a study in which he applied circumostial Mitomicin C during external and endoscopic DCR with an anatomical success rate of 97.3% and a functional success rate of 96.4% [**40**].

**Table 3:** Success rate of internal and external DCR [**34**-**37**]

	Success rate	
	External DCR	Internal DCR
Hartikainen J (1998)	89.1%	90.2%
Cokkeser Y (2000)	88.2%	89.2%
Vivek KP (2013)	90%	86.67%
Dey AK (2014)	93.33%	90%

## Conclusions 

During the last years, many trials regarding congenital nasolacrimal duct obstruction have been published, making it possible to conclude that the first treatment choice in uncomplicated cases should be a conservative treatment which can be applied until the age of 1 year. In complicated cases, a complex management should be taken into consideration.

In the last years, new techniques have been developed and classic techniques have been improved, which enhances the outcome of CNDO.

**Disclosure**

None.
